# Development of the Diet Quality Questionnaire for Measurement of Dietary Diversity and Other Diet Quality Indicators

**DOI:** 10.1016/j.cdnut.2024.103798

**Published:** 2024-06-21

**Authors:** Anna W Herforth, Terri Ballard, Andrew Rzepa

**Affiliations:** 1 Department of Global Health and Population, Harvard T.H. Chan School of Public Health, Boston, MA, United States; 2 Independent Scholar, Rome, Italy; 3 Gallup, Inc., Dubai, United Arab Emirates

**Keywords:** nutrition surveillance, dietary assessment, healthy diets, WHO guidelines, food-based dietary guidelines, food systems, Sustainable Development Goals

## Abstract

**Background:**

To monitor trends toward healthy and sustainable diets, there is a need for feasible survey tools, with cross-cultural validity, low-cost, and low-expertise requirements.

**Objectives:**

The objective of this research was to develop a method to gather data suitable for monitoring diet quality in the general population (women and men of all ages) that is feasible within multitopic surveys, low burden for both enumerators and respondents, valid at population level, and that captures the information necessary for understanding diet quality at global and local levels.

**Methods:**

A literature review was conducted to identify constructs of diet quality with existing consensus, indicators with existing global demand, and methods that may be feasible and valid. Results were presented to a technical advisory group for debate, resulting in consensus on a set of constructs to be measured, desired indicators, viable data collection platforms, and an approach for testing and piloting.

**Results:**

Food group-based indicators and 24-h recall period were selected as the most feasible and valid approach for population-level monitoring. A 29-item Diet Quality Questionnaire (DQQ) was developed, where each yes/no question asks about the consumption of a distinct food group on the previous day or night. The food groups were selected for the purpose of deriving indicators to capture the constructs for which there was consensus: nutrient adequacy, and protection against noncommunicable diseases, including both positive and negative risk factors.

**Conclusions:**

The DQQ is low cost and feasible to administer in existing large-scale surveys, overcoming barriers to diet data collection that have precluded the routine monitoring of diet quality in the past. This novel approach has now been used across >85 countries in the Gallup World Poll and other surveys, generating the first nationally representative available datasets on Minimum Dietary Diversity for Women and complementary diet quality indicators.

## Introduction

Diet quality is one of the most important public health issues – yet it has not been measured in the general population within most countries or across countries. Poor diets and malnutrition are the greatest risk factors globally for deaths and disability-adjusted life-years lost [1]. Inadequate diets are also a direct contributor to child undernutrition [2], which causes 45% of child deaths [3]. Routinely collected, globally comparable information on diet quality is needed to understand dietary trends critical for public health and food systems transformation for healthy and sustainable diets. The lack of dietary data has until now precluded the inclusion of a diet quality indicator in the Sustainable Development Goals (SDG).

There are many reasons dietary data – information on what individuals actually eat or drink – have not been collected across countries globally before. One is that traditionally, national nutrition surveys or national food consumption surveys have been standalone survey efforts. These surveys are expensive and require bespoke infrastructure, training, and budgets. Furthermore, they must be administered by highly trained nutrition professionals, and data analysis is complex and often takes several years. The fiscal and expertise requirements of standalone nutrition surveys have usually not been feasible to prioritize in the budgets of low- and middle-income countries (LMIC), and even many high-income countries (HIC). So dietary data, where it exists, have been patchwork and piecemeal; in some countries, dietary data have been gathered sporadically in ways that are either very comprehensive and expensive (e.g., weighed food records and quantitative 24-h recall); or in ways that are very light and lack essential information to describe diets as a whole (e.g., questions about fruit and vegetable consumption habits [4,5]).

Even if sufficient funds were available in every country, other barriers exist. A set of simple indicators has not been defined at the global level for diet quality in the general population (women and men of all ages). Many diet quality indicators for the general population have been developed, but few have demonstrated global cross-cultural validity, utility, and feasibility in global survey mechanisms [6,7]. Even more fundamental than a set of indicators, is the clarity on a core set of constructs or principles that make-up diet quality and are universally relevant. The need for consensus on constructs and indicators of diet quality has been voiced [7].

Indicators have been developed for subpopulations, including infants and young children aged 6–23 mo, and women aged 15–49 y. Infant and young child feeding (IYCF) indicators have been defined in several iterations, most recently by WHO and UNICEF (2021) [8,9], collected across countries in the Demographic and Health Surveys (DHS) and Multiple Indicator Cluster Surveys (MICS), and made available by UNICEF [10]. The first indicator with wide endorsement for use in adult populations in LMIC globally was the Minimum Dietary Diversity for Women (MDD-W), a proxy indicator of nutrient adequacy among women aged 15–49 y [11,12]. It has recently been proposed as an indicator in the SDGs, demonstrating that it is widely considered to be useful. It is also widely recognized, however, that the MDD-W is not an indicator of total diet quality [6,13]. It does not correspond well to diet-related noncommunicable disease (NCD) risk factors [14], and it also has not been validated for use among men or older women. These gaps could be filled with other complementary indicators for the total population. Furthermore, although data collection methods for MDD-W require only food group consumption data, classifying foods into food groups and adapting questions to local contexts presented challenges for nonnutritionists to carry out independently, hindering scaled data collection across countries.

The Global Diet Quality Project was initiated with the aim of diet quality monitoring within and across countries. This aspiration was informed by the authors’ involvement and experience in 3 advances in global food measurement that culminated around 2014. The first was the establishment of global data collection for the Food Insecurity Experience Scale (FIES) [15]. The Gallup World Poll (GWP) survey platform played a catalytic role in FIES data collection in >140 countries, enabling 2 outcomes: the establishment of a FIES-based SDG indicator due to the availability of data, which is a prerequisite for global monitoring indicators; and the availability of FIES survey tools that countries could implement in their own diverse surveys. In the decade since FIES measurement began in the GWP, 60 countries have now incorporated the FIES into their national surveys [16]. We wondered whether it would be possible to design a diet quality module that could be scaled up and implemented globally in a similar way.

Second, new indicators of IYCF were being developed in a consultative process at the same time as the Global Diet Quality Project was initiated. That was relevant because the new indicators signaled consensus in the nutrition community, taken up by United Nations (UN) institutions, on aspects of diet quality that may be shared across the life cycle. The new indicators included food group-based indicators of healthy practices (≥1 vegetable or fruit; ≥1 flesh food or egg), and unhealthy food consumption (sweet beverages and sweet or savory unhealthy sentinel foods). They also demonstrated that a complex set of practices around infant feeding could be captured by a suite of indicators rather than just 1 and that individual aspects of feeding (such as dietary diversity, breastfeeding, and meal frequency) were often more informative when reported separately than in a single indicator (e.g., minimum adequate diet) [8]. Furthermore, they demonstrated that as knowledge and practice evolve, indicators can be updated. We learned from the IYCF indicator development process, seeing a suite of indicators including healthy and unhealthy practices, from underlying data that allow indicator calculation to evolve.

Third, the development of the MDD-W, and its acceptance in 2014 [17], was a blueprint for the development and measurement of additional diet quality indicators. The MDD-W resulted from over a decade of concerted thinking and research toward a practical, meaningful indicator across settings that had vastly lower data requirements than pre-existing methods [11,12,[18], [19], [20], [21], [22], [23]]. The core innovation of the MDD-W was using food groups to proxy for quantitative dietary intake. We built on that thinking, adding other food group-based indicators focused on NCD risk factors, to complement the MDD-W [13].

The Global Diet Quality Project sought to develop a survey module to measure diet quality in a way that is feasible and valid to implement in the GWP or other large-scale surveys, and that would capture the most essential pieces of information for understanding and monitoring diet quality across countries. This information would be useful for constructing multiple population-level diet quality indicators, for the purpose of global, national, and subnational tracking of levels and improvements or deteriorations in diet quality. This paper reports the results of the process to develop the questionnaire.

## Methods

The process to develop a diet quality survey module was done in 3 parts: a literature review, a technical advisory group (TAG) meeting, and a design and piloting process reviewed by the TAG. The literature review was conducted to identify constructs of diet quality with existing consensus, indicators with existing global demand, and methods that may be feasible and valid. This review focused on statements or indicators from normative global agencies, national governments, and large global studies, *1*) to identify the aspects of diet quality that should be measured for global monitoring based on existing consensus, *2*) to identify areas where there is not a strong case for monitoring based on lack of consensus, and *3*) to explore possible approaches to designing a questionnaire that would collect the right data for the desired constructs and would fit the parameters of foreseeable data collection platforms. Then, the review article [24] was presented to a TAG as background for discussion about objectives, indicators, and methods for assessing diet quality through a survey module. The TAG was convened by Gallup and composed of dietary assessment experts, and included the directors and supporting staff of the UN FAO nutrition division, WHO nutrition division, bilateral organizations that measure diet, donors interested in diet quality measurement, and academia [25]. The goal of global monitoring at scale required balancing ideals with real-world constraints. The TAG, convened in 2016, sought to answer the following questions:

1. What constructs of diet quality should be measured?
2. Are existing indicators sufficient to measure them?
3. What data collection platforms could be used for diet data collection at scale?
4. What methods should be used to collect data for population-level monitoring?

The consideration of these questions was based on utility, feasibility, and validity in equal shares. Utility refers to capturing important information for understanding diets and their variation across contexts. Feasibility refers to the reality that no data collection will happen unless it fits within the constraints and parameters of existing data collection platforms. Validity is considered at the population level because the purpose is not for individual assessment – it is not expected that the tool be sensitive enough to use for targeting or diet counseling, but rather for population-level statistics.

Following the recommendations of the initial meeting, a process was undertaken to draft a Diet Quality Questionnaire (DQQ), identify relevant food groups, and refine questions on the basis of the insights from quantitative dietary intake data from 2 countries, Brazil and the United States. The TAG then reconvened in 2018 to review a proposed methodology and its pilot results, including decisions around the design of a food group-based module. This manuscript summarizes the outcomes of the discussion article [24], the TAG meeting report [25], and the basis for food group selection; and how these shaped the design of the DQQ that has now been implemented across 85 countries [26].

## Results

The TAG strongly endorsed the value of monitoring dietary quality globally. Poor dietary intake is a cause of malnutrition in all its forms, and global data are needed to support an improved evidence base of how dietary trends are related to nutritional status and health outcomes, and to food systems transformation. Furthermore, the consensus was that monitoring should not seek comprehensive measurement of the total diet; rather it must capture indicators of the components of diet that have the greatest impact on health and nutrition across and within contexts. Indicators would be used for monitoring trends, informing policy action, and providing evidence for large-scale efforts of food, agriculture, and nutrition policies.

### What constructs of diet quality should be measured?

The TAG emphasized the following 2 key aspects of diet quality: *1*) nutrient adequacy and *2*) protection of health against NCDs. These are universally important across regions and countries, including those in which diets are rapidly transitioning, which a global diet quality module should seek to measure. They reflect the WHO Healthy Diet Fact Sheet (2018) [27]: “A healthy diet helps to protect against malnutrition in all its forms, as well as noncommunicable diseases (NCDs) such as diabetes, heart disease, stroke, and cancer”; and “The exact make-up of a diversified, balanced and healthy diet will vary depending on individual characteristics (e.g. age, gender, lifestyle, and degree of physical activity), cultural context, locally available foods, and dietary customs. However, the basic principles of what constitutes a healthy diet remain the same.” These concepts were echoed in a recent consensus document on healthy diet monitoring, with key constructs referred to as adequacy, diversity, and moderation [7]. Diversity in food groups is important for nutrient adequacy, and diversity of plant foods specifically (that contain antioxidants, fiber, and other health-promoting compounds) is important for NCD protection; and moderation or avoidance of risk factors for NCDs (e.g., excess free sugar, salt, and nitrates) also protects against NCDs. In our 2016 TAG, as in later expert groups, the environmental impact of diets was a construct that the TAG recommended would be ideal to measure, but that was not integral to diet quality itself. Table 1 [[27], [28], [29], [30], [31], [32], [33], [34], [35], [36], [37], [38], [39], [40], [41]] shows a summary of the literature review of global and national dietary recommendations, research, and indicators related to these constructs [24].

**TABLE 1:** Aspects of diet quality identified in global and national dietary recommendations and measured as risk factors in global diet-disease research.

Construct	Dietary recommendation or risk factor	WHO healthy diet^1^	National FBDGs^2^	GBD risk factor^3^	Measured in diet quality indexes designed for international use^4^	Sustainable diets^5^
Nutrient adequacy	Dietary diversity	x	x		(Some)	x
Protection against NCDs	Fruits and vegetables	x	x	x	x	x
	Whole grains	x	x	x	(Some)	x
	Legumes, nuts and seeds	x	(Some)	x	x	x
	Dietary fiber	x	x	x	(Some)	
	Fish		(Some)	(Omega-3)	(Some)	
	Dairy			x	(Some)	(Reduce)
	PUFA			x	(Some)	
Increased risk of NCDs	Excess sugar	x	x	(SSB)	x	(The principle of limiting UPF intake relates to several risk factors closely associated with UPF intake)
	Excess salt	x	x	x	x	
	Excess fat	x	x		(Some)	
	Excess saturated fat	x	x		(Some)	
	Trans fat	x	(Some)	x	(Some)	
	Processed meat	x	(Some)	x	(Some)	
	Excess red meat	x	(Some)	x	(Some)	x

Abbreviations: AHEI, Alternative Healthy Eating Index; DASH, dietary approaches to stop hypertension; DQI-I, Diet Quality Index International; FBDG, food-based dietary guidelines; GBD, global burden of disease; GDQS, Global Diet Quality Score; HEI, Healthy Eating Index; IARC, International Agency for Research on Cancer; NCDs, noncommunicable diseases; MDS, Mediterranean diet score; SSB, sugar-sweetened beverages; UPF, ultraprocessed food.

An “x” denotes the dietary factor as a recommendation or risk factor included in the type of document noted.

Source: Adapted from Herforth, 2016 [24]

1 Recommendations and guidelines from WHO [[27], [28], [29], [30], [31]], other than risk factors (processed meats) from WHO IARC [32].

2 Summarized from the conclusions of Herforth et al. [33], a global review of FBDG for 93 countries.

3 Risk factors identified by the Global Burden of Disease Study [1,34] and the Global Dietary Database.

4 Diet quality indexes constructed for international use, from quantitative or semiquantitative data, including AHEI, HEI, DASH, DQI-I, GDQS, and MDS [[35], [36], [37], [38], [39], [40]]. An “x” means that the dietary risk factor is included in all of these indexes.

5 Source: FAO and WHO, 2019 [41].

### Are existing indicators sufficient to measure the most important aspects of diet quality?

The TAG recommended aiming for a dashboard of indicators, each capturing a different construct. Separate indicators could possibly later be combined into an index, but retaining them individually would be important for clear understanding of the different aspects of diet quality in each place, given that they could move in opposite directions (i.e. dietary diversity could improve concomitantly with increased consumption of sugary, salty, and fatty foods). This follows the model of IYCF indicator development.

For the construct of nutrient adequacy, the TAG endorsed the use of MDD-W as a proxy measure of nutrient adequacy. Although the dichotomous indicator is validated only for women aged 15–49 y, dietary diversity scores can be used as an indicator of micronutrient-rich food consumption for the general population [42].

For the construct of protection against NCDs, no indicators were identified that could be adapted to the type of data collection platform available. There are many existing indicators [[35], [36], [37], [38], [39],^43^,^44^], but these require quantitative data that cannot be feasibly collected within multitopic surveys [6,45]. The MDD-W, which is feasible, does not correspond well to NCD risk [14]. The TAG concluded that there was a gap and a need for new low-burden indicators of diet quality related to NCD risk. Subsequently, the indicators “NCD-Protect” and “NCD-Risk” were developed from food group data to meet this charge [13]. The new indicators were based on WHO recommendations related to the prevention of NCDs [27,28,32].

There was discussion about using an indicator of ultraprocessed foods (UPF) consumption as a marker of dietary shifts related to the nutrition transition, and as a proxy measure for both lower micronutrient intakes and higher risk of obesity and NCDs [[46], [47], [48], [49], [50]]. Although the new indicator validation was based on WHO recommendations rather than UPF consumption per se, lower adherence to WHO recommendations and UPF consumption are correlated, so the new indicators (especially NCD-risk) are empirically correlated with UPF consumption [13].

Indicators of environmental impact were also considered, but were considered to be a lower priority for immediate development; nonetheless, relevance for environmental impact was a factor in selecting the food groups included in the DQQ.

### What data collection platforms could be used for diet data collection at scale?

A viable data collection platform is essential for the feasibility of global monitoring of diet quality. Creating a new survey platform for a diet survey would be much more costly (and in some contexts infeasible) to build than integrating a module into existing multitopic surveys. Fortunately, several multitopic surveys cover nationally representative samples of the adult population in multiple countries, for monitoring of other global indicators. Different platforms have different strengths and limitations.

The Gallup World Poll (GWP) is a yearly survey that interviews a representative sample of 98% of the world’s population in >140 countries annually since 2006. GWP is a unique platform in that it covers all countries where surveys are feasible and allowed by governments, including HIC. It covers the total population aged ≥15 y, which enables the assessment of gender differences. Additionally, data are collected in nationally representative samples annually, and results are available rapidly, 2–4 mo after data collection ends. GWP has demonstrated the capability of monitoring SDG indicators that are accepted by global institutions and national governments, including the FIES. The main limitations of data collection through GWP are sample size, and data collection at a single time point. Sample sizes are relatively small (usually 1000 per country) and therefore can only be disaggregated with limited descriptive variables. Regional or state-level estimates are not possible. Measurement at one time point will not be representative of the whole year in places where diets vary by season.

The Demographic and Health Surveys (DHS) are conducted every 5 y in ≤94 LMIC. DHS have been a major source of data as a routine multitopic survey with country ownership. DHS typically has a large sample size and covers several months of the year, offering important benefits for analysis by subnational region and potentially by season. Limitations of DHS are that it does not cover HIC, has relatively infrequent survey cycles (some countries participate sporadically in each 5-yearly cycle), and that currently in most countries it is administered only among 1 adult demographic group (women aged 15–49 y), limiting the ability for assessing gender and age differences in diet. Analysis and data release usually take a year or more.

The Living Standards Measurement Study (LSMS) and other household consumption and expenditure surveys (HCES) are a heterogeneous class of multitopic surveys. They are designed by, or in close collaboration with, national statistical offices, and can cover a variety of topics of interest to national stakeholders. They have many strengths: coverage of large samples that are nationally representative and often regionally representative over several months, offering benefits for analysis by region and potentially by season, as well as enabling research on the associations between diet quality and other household variables. In countries where the FIES module has been adopted, it has been implemented in HCES as well as other country-owned multitopic surveys (such as national health surveys or agriculture censuses) [51]. A similar model could serve to be useful for the country’s uptake of a diet quality survey module.

The GWP provided useful boundaries of the questionnaire development, because national surveys (such as HCES, national health surveys, or agriculture censuses) face similar constraints: covering many topics, needing to minimize time on any 1 module to minimize respondent fatigue and keep costs feasible, not necessarily being conducted in person, and not typically having trained nutritionists as enumerators.

### What methods should be used to collect data for population-level monitoring?

A module more than a few minutes long or with special equipment or training needs would not be feasible to scale up within existing national survey platforms. Concerns about time and respondent fatigue are paramount. Quantitative 24-h recalls, although useful for many reasons, would not be feasible for diet quality monitoring at scale. That type of data is far too costly, requiring specific expertise and a high time burden of data collection and analysis. Even semiquantitative data using any kind of visual aids would increase requirements beyond the threshold of feasibility in available survey platforms. The survey module must also be possible to be administered by telephone, the mode of GWP data collection in many countries, precluding visual aids.

Several possible approaches to gathering diet quality data include the following:

• nonquantitative 24-h recall: open recall,
• nonquantitative 24-hour recall: "list-based" questions about food groups consumed in the previous day,
• food frequency questionnaires (FFQ),
• Likert scale questions about typical consumption (e.g. never, sometimes, often, and always),
• questions about preferences, and
• other new methods.

The TAG concluded that a nonquantitative 24-h recall would be the best type of method for monitoring. In nutrition assessment, 24-h recalls or food records are generally the preferred methods for characterizing the diets at the population level. Although a single 24-h recall does not provide a valid assessment of an individual’s diet, it does provide a valid cross-sectional characterization of a population [[52], [53], [54]]. Although stated preferences correlate with actual consumption [55], questions about preferences were eliminated as proxies for diet quality because they were not likely to be precise enough, or equally valid across low- and high-income contexts. Likert scale questions about typical consumption have a risk of social desirability bias, and experiential evidence from GWP on other topics showed that Likert scales were not highly comparable across different education and ethnic backgrounds. An abbreviated FFQ, such as a “7-day recall,” was considered. Respondents cannot actually recall intake for a whole week, however, so the cognitive process for this recall period requires “generalized” rather than specific memory, quite similar to a Likert scale of usual consumption. FFQ is less preferred for population-level monitoring and demands a higher cognitive burden on respondents compared with a 24-h recall [54]. Other newer methods were considered, such as using mobile phones to take photographs of food [56], but these methods are not yet validated across settings nor currently amenable to existing survey platforms.

There are 2 methods for the nonquantitative 24-h recall, open recall (where an enumerator asks the respondent to list everything consumed in the previous day or night, which is then categorized into food groups) or list-based questions (asking the respondent if they have consumed a set of foods or a food group in the previous day or night). Nguyen et al. [57] assessed the relative validity of the list-based and open recall methods, compared with a quantitative 24-h recall, and concluded that both methods performed similarly and adequately to predict micronutrient adequacy for women in 2 South Asian settings; Hanley-Cook et al. [58] compared both methods with a weighed food record in several countries and came to similar conclusions. After assessing the training and data processing needs of each, Gallup determined that an open recall would be infeasible, because it would require specific training as well as special software to record items consumed, and in-depth local expertise to categorize each food into food groups, with the possibility of missing data for unknown foods, such as those reported using a local linguistic dialect. Therefore the list-based method was selected. This was the same conclusion of the DHS-8, which started including data collection for MDD-W in its core module in 2020, using the list-based method [59].

### Food group selection necessary for the indicators and constructs

Given that the desired tool would be a list-based 24-h recall, the next step was to select food groups necessary for constructing indicators of nutrient adequacy and protection of health against NCDs. On the advice of the TAG, the DQQ was built on the MDD-W questionnaire [11,12]. The DQQ uses the food groups used for gathering data on MDD-W and further disaggregates them, ultimately including 29 food groups to meet multiple purposes (Table 2): *1*) data suitable for computing MDD-W, aligned with the measurement guide published by FAO, *2*) alignment with new IYCF indicators published by WHO and UNICEF, *3*) sufficient information for new indicators of NCD risk factors that could reflect WHO healthy diet recommendations, drawing upon existing literature and indicators such as the Global Diet Quality Score (GDQS), *4*) ability to track UPF consumption trends, and *5*) relevance to environmental impact of diets. The overall objectives were alignment with existing guidance from the global normative agencies, and parsimony: the minimum number of food groups necessary for the maximum useful information to meet these purposes. Subsequent analysis of correlations with quantitative dietary factors helped to provide further evidence for choice of the 29 food groups [13].

**TABLE 2:** The 29 food groups of the DQQ and rationale for their inclusion and the level of disaggregation.

	Food groups^1^	Source and rationale for disaggregation beyond the aggregate food groups used in MDD-W and IYCF indicator calculation
	**Grains, white roots and tubers, and plantains**	MDD-W [12]
1	Foods made from grains	Cognitive ease, more manageable questions; disaggregated in MDD-W example questionnaire [12]
2	White roots, tubers, and plantains	
3	Whole grain foods	Globally recommended for protection of health against NCDs [27]
4	**Pulses/legumes (beans, peas, and lentils)**	MDD-W [12]
5	**Nuts and seeds**	MDD-W [12]
	**Milk and milk products**	MDD-W [12]
6	Fluid milk	*1*) Cognitive ease, more manageable questions, *2*) alignment with disaggregation in WHO and UNICEF 2021 [8], and *3*) differential environmental impacts
7	Cheese	
8	Yogurt	
	**Meat, poultry, and fish**	MDD-W [12]
9	Processed meats	*1*) Cognitive ease, more manageable questions; recommended disaggregation in MDD-W example questionnaire [12], *2*) differential health impacts, *3*) differential environmental impacts, *4*) information of possible interest to data users, as the food system determinants of each subgroup differ greatly
10	Unprocessed red meat (ruminant)	
11	Unprocessed red meat (nonruminant)	
12	Poultry	
13	Fish and seafood	
14	**Eggs**	MDD-W [12]
15	**Dark green leafy vegetables**	MDD-W [12] Note: in the DQQ, this food group is sometimes asked with 2 questions to capture all common items in the food group, which are then aggregated at the analytical stage.
	**Other vitamin A-rich fruits and vegetables**	MDD-W [12]
16	Vitamin A-rich orange vegetables	*1*) Cognitive ease, more manageable questions; disaggregated in MDD-W example questionnaire [12], *2*) used separately in indicators on total vegetable consumption, and total fruit consumption
17	Vitamin A-rich fruits	
18	**Other vegetables** (i.e., vegetables that are not orange or dark green leafy)	MDD-W [12] Note: in the DQQ, this food group is sometimes asked 2 questions to capture all common items in the food group, which are then aggregated at the analytical stage.
	**Other fruits**	MDD-W [12]
19	Citrus	*1*) Easier to capture all common items if disaggregated into 2 questions, *2*) indicators with 3 instead of 2 fruit subgroups proved to have a stronger correlation with total fruit intake [13], and *3*) citrus is an easy-to-identify subgroup, common in all countries, and has been suggested to have a unique role in NCD prevention [14].
20	Other fruits (i.e., fruits that are not citrus or vitamin A-rich)	Note: in the DQQ, this food group is sometimes asked with 2 questions to capture all common items in the food group, which are then aggregated at the analytical stage.
	**Sweet beverages**	WHO and UNICEF 2021 [8], FAO 2021 [12]
21	Sweet tea/coffee/cocoa	*1*) alignment with disaggregation in the example questionnaire of WHO and UNICEF 2021 and *2*) indicators with 3 sweet beverage subgroups proved to have a stronger correlation with free sugar intake than if the groups are aggregated [13]
22	Fruit juice and fruit-flavored drinks	
23	Soft drinks (sodas, energy drinks, and sports drinks)	
	**Sweet foods**	WHO and UNICEF 2021 [8], FAO 2021 [12]
24	Baked/grain-based sweets	*1*) Easier to capture all common items if disaggregated into 2 questions and *2*) indicators with 2 sweet food subgroups proved to have a stronger correlation with free sugar intake than if the groups are aggregated [13]
25	Other sweets	
	**Fried and salty foods**	WHO and UNICEF 2021 [8], FAO 2021 [12]
26	Packaged ultraprocessed salty snacks	*1*) Easier to capture all common items if disaggregated into subquestions; FAO 2021 suggests collecting these disaggregated food groups along with MDD-W [12]; *2*) indicators with 4 salty/fried snacks subgroups proved to have a stronger correlation with ultraprocessed foods and WHO recommendations than if the subgroups are aggregated [13], *3*) information of possible interest to data users, as the food system determinants of each subgroup differ
27	Instant noodles	
28	Deep-fried foods	
29	Fast food	

Abbreviations: DQQ, Diet Quality Questionnaire; FAO, Food and Agriculture Organization of the United Nations; MDD-W, Minimum Dietary Diversity—Women; NCDs, noncommunicable diseases.

1 Food groups used in MDD-W [12] and IYCF [8] indicator calculation are displayed in bold text.

The new IYCF indicators included an indicator on "zero vegetable or fruit consumption", sweet beverage consumption, and unhealthy food consumption (sentinel sweet unhealthy foods and sentinel salty/fried unhealthy foods) as indicators of unhealthy practices [8]. In a diet quality monitoring system, ideally, the same or similar information is gathered across the life course. This is especially pertinent for DHS, which is implementing questionnaires for IYCF and for women, asked of the same individual. It is easier for the respondent and less confusing if the information asked is aligned. Furthermore, it allows the computation of comparable indicators that can be compared between adults and children, which can help illuminate causes of poor diet quality or intervention impact on the basis of better information of intrahousehold disparities [60]. We therefore included the same food groups newly included in IYCF surveys.

The DQQ does not include 3 optional food groups that are often collected alongside the MDD-W. These are organ meats, which are aggregated into red meat and poultry questions, red palm oil, and insects and other small protein foods. These food groups are not used in the calculation of MDD-W or any indicator related to NCDs. Therefore, they were excluded from the DQQ on grounds of parsimony. These food groups are used in the DHS, however, and country-adapted forms of each question can be added based on user interest.

Diversity in fruit and vegetable consumption was of interest because consumption of diverse plant foods might provide greater NCD protection [61,62], and because diversity in fruits and vegetables could be explored as a proxy for quantitative intake of the same. Although the vegetable group is already disaggregated into 3 subgroups in the MDD-W, the fruits group had only 2 subgroups. Early in DQQ development, an additional food group of “red/blue fruits” was considered, as a way to capture botanical variety in phytochemicals. However, this category was dropped because of the finding that in many countries, few red or blue fruits are consumed, so its cross-cultural relevance for global indicators was limited. Citrus was added instead, following its use in another indicator validated for NCD protection, Prime Diet Quality Score (PDQS, later developed into the GDQS) [14,40]. In addition to having some evidence of unique association with NCDs, it served the purpose of globally relevant disaggregation to collect information on the diversity of fruits consumed, because citrus is found as a commonly consumed type of fruit in every country.

Additional food groups included in PDQS/GDQS were closely considered, given the similarity of constructs measured [40]. Where the DQQ food groups differ from GDQS, the difference is intentional. Unsaturated oils (a food group in GDQS) were of interest based on consensus around healthy compared with unhealthy fats in NCD prevention. However, most respondents cannot identify the type of oil they consumed unless they cooked all of their own food. Therefore, “type of oil” was considered desirable to measure, but not valid in a survey setting. Low- and high-fat dairy (2 food groups in GDQS) are also not separated in the DQQ because respondents in most settings cannot reliably identify whether they consumed low-fat or high-fat dairy. Cruciferous vegetables (a food group in GDQS) were not added to the DQQ because that category overlaps with 3 different food groups used in MDD-W (dark green leafy vegetables, white roots, and other vegetables) [12], thereby disrupting the utility of the data for calculating MDD-W.

Some specific categories of UPF were included because they appear around the world as food systems change. These include packaged ultraprocessed salty snacks, instant noodles, fast foods, soft drinks, and fruit drinks. These serve as bellwethers of the nutrition transition and contribute to an indicator tracking UPF consumption [13]. To build a forward-looking questionnaire related to the environmental impact of diets, the animal-source food groups were disaggregated into subgroups with differential associated greenhouse gas emissions and other impacts [63].

## Discussion

Significant consensus exists in the international community on the most important and universal aspects of a healthy diet. This consensus can be leveraged for the measurement of dietary quality and can be operationalized through food group-level data to produce indicators aligned with existing documents published by global and national institutions. What our 2016 TAG identified has been echoed in other subsequent expert groups on diet quality [7,64].

### Steps taken after the DQQ was developed

The final outcome of the TAG meeting was to determine the next steps for the design, testing and validation of a survey module suitable for global implementation. These conclusions were the roadmap for all the work to develop, adapt, and validate the DQQ. Subsequent work was done to adapt the questionnaire for each country; to conduct cognitive testing and a quantitative pilot test in a multitopic survey setting; and to conduct validation studies of the DQQ against quantitative standard methods. Methods and results from these studies are reported elsewhere [13,26,65]. An indicator calculator is available to automate the analysis of DQQ data [26]. The DQQ has been implemented now in 85 countries in the GWP, and each has supplied the country-adapted questions implemented in the DHS-8 for the measurement of MDD-W and IYCF indicators [66]. The DQQ has also been implemented in the Ethiopia and Nigeria LSMS and is in use to evaluate MDD-W within the Feed the Future program [67].

### Limitations

The DQQ has limitations. It is for population-level diet quality assessment and is not designed or valid for clinical assessment of individuals. It does not assess dietary energy (a notoriously difficult indicator, captured poorly even in quantitative surveys), nor does it assess quantitative intakes of nutrients or foods (except via proxy indicators based on diversity). Thus, it does not replace 24-h recall or other quantitative surveys. Quantitative data are needed for applications such as understanding trends and disparities across populations in amounts of foods or beverages consumed or design of fortification programs. Although DQQ data can pick up the change in prevalence of consumption of a certain food group or beverage (such as in response to a tax on soft drinks), they cannot show the change in quantities consumed (e.g., if soft drink quantity of consumption shifted, without a change in prevalence). When using the DQQ for program evaluation, like any data collection tool, implementers will have to think through what they need to measure; the DQQ does not assess all things that may be of interest for diet quality, especially as related to some agriculture interventions such as biodiversity in food intake. Additional questions could be added when using the DQQ in the context of program evaluations. For example, for those interested in biodiversity: for food groups where the respondent answered yes, it is possible to follow up with additional questions about what items were consumed, at whatever level of detail the researcher desires and has the capacity to measure, e.g. at species or cultivar level.

### Strengths

Although inherently limited, as a 5-min survey module, the DQQ has many strengths. It costs <1% of a quantitative dietary intake survey; in-country use of the DQQ has been done for ∼$1 per respondent [68], whereas quantitative 24-h recalls in LMIC cost on the order of $500–800 per respondent [69]. Although the DQQ does not provide all the rich detailed information of a quantitative survey, at ∼500 times lower cost, the information: cost ratio would seem quite high for the DQQ. Inserting the DQQ within a multitopic survey requires no extra topic-specific training for either data collection or analysis; analysis of DQQ data is rapid and automated. Thus it overcomes major barriers to diet data collection. On the basis of careful consideration of data needs, the DQQ builds on the MDD-W and maximizes the information obtained from a short set of questions. It provides a suite of indicators capturing the constructs where there is broad consensus – nutrient adequacy and protection against NCDs, including both protective factors and risk factors. The 29 food groups can be adapted to produce other indicators of interest, such as adherence to dietary guidelines or the environmental impact of diets.

Additional strengths have to do with the process to develop, adapt, and implement the DQQ. Based on input from the UN agencies at the beginning of the design process, the DQQ captures the constructs and indicators where there was pre-existing consensus, including all the indicators and recommendations published by the global normative agencies on diet and nutrition: WHO [[27], [28], [29], [30],^32^,^63^], UNICEF [8], and FAO [12,16]. The approach was intentionally designed with input and collaboration from each of these institutions, starting with the first TAG meeting. With a long view of data suitable for SDG indicators, the emphasis on global normative institutions was intentionally pursued for acceptability among member states. Letters of support from the nutrition directors of WHO, FAO, the United Nations World Food Programme, and regional UNICEF staff increased the willingness and trust of hundreds of key informants around the world to participate in questionnaire adaptation.

Additionally, the DQQ was subsequently adapted with input from DHS and Feed the Future, which are now using the DQQ questions to collect MDD-W data [67]. The DQQ food groups allow for data collection aligned between IYCF and adults, which is a strength that allows easier data collection, analysis, and interpretation across the life course to better understand determinants of malnutrition. Ongoing communication with LSMS is uncovering new learnings from implementation in individual country surveys. These partnerships were intentionally pursued, as extremely important for alignment across the major surveys to avoid fragmentation and confusion in data collection efforts.

### Conclusion

The approach to design the DQQ has been done to enable monitoring of diet quality within and across countries, using a method that is feasible at scale. The DQQ can be used to monitor progress annually at a national, regional, or global level. The indicators generated are aligned with the work of global normative agencies to characterize diet quality at the population level. This approach elevates visibility around healthy diets and helps encourage diet quality monitoring via existing data collection platforms in countries around the world.

## Acknowledgments

We acknowledge Lawrence Haddad and Patrick Webb who worked with AR to draft the initial concept note for the TAG, and for sparking early conversations about the need for this agenda. We thank Pablo Diego Rosell and Taylor Auger at Gallup for initial analytical and organizational inputs, and Zacc Ritter and two anonymous reviewers for helpful comments on the manuscript.

## Author contributions

The authors’ responsibilities were as follows – AH, TB, AR: designed research; AH, TB: conducted research; AR: provided essential materials; AH: wrote the paper and had primary responsibility for final content; and all authors have read and approved the final manuscript.

## Conflict of interest

The authors report no conflicts of interest.

## Funding

Funding for the work described in this manuscript was provided by the Swiss Agency for Development and Cooperation (SDC).

## Data availability

Data described in the manuscript (discussion paper and meeting report) is publicly and freely available without restriction at dietquality.org.
